# Bioactive peptides: an alternative therapeutic approach for cancer management

**DOI:** 10.3389/fimmu.2024.1310443

**Published:** 2024-01-24

**Authors:** Nooshin Ghadiri, Moslem Javidan, Shima Sheikhi, Özge Taştan, Alessandro Parodi, Ziwei Liao, Mehdi Tayybi Azar, Mazdak Ganjalıkhani-Hakemi

**Affiliations:** ^1^ Department of Immunology, Faculty of Medicine, Ahvaz Jundishapour University of Medical Sciences, Ahvaz, Iran; ^2^ Department of Immunology, Faculty of Medicine, Isfahan University of Medical Sciences, Isfahan, Iran; ^3^ Department of Food Engineering, Faculty of Engineering, Yeditepe University, Istanbul, Türkiye; ^4^ Scientific Center for Translation Medicine, Sirius University of Science and Technology, Sochi, Russia; ^5^ Department of Hematology, Guangzhou Women and Children's Medical Center, Guangzhou Medical University, Guangzhou, China; ^6^ Department of Biophysics, Faculty of Medicine, Yeditepe University, Istanbul, Türkiye; ^7^ Regenerative and Restorative Medicine Research Center (REMER), Research Institute for Health Sciences and Technologies (SABITA), Istanbul Medipol University, Istanbul, Türkiye

**Keywords:** anticancer, bioactive peptide, cancer therapy, immunomodulation, cancer immunotherapy

## Abstract

Cancer is still considered a lethal disease worldwide and the patients’ quality of life is affected by major side effects of the treatments including post-surgery complications, chemo-, and radiation therapy. Recently, new therapeutic approaches were considered globally for increasing conventional cancer therapy efficacy and decreasing the adverse effects. Bioactive peptides obtained from plant and animal sources have drawn increased attention because of their potential as complementary therapy. This review presents a contemporary examination of bioactive peptides derived from natural origins with demonstrated anticancer, ant invasion, and immunomodulation properties. For example, peptides derived from common beans, chickpeas, wheat germ, and mung beans exhibited antiproliferative and toxic effects on cancer cells, favoring cell cycle arrest and apoptosis. On the other hand, peptides from marine sources showed the potential for inhibiting tumor growth and metastasis. In this review we will discuss these data highlighting the potential befits of these approaches and the need of further investigations to fully characterize their potential in clinics.

## 1 Introduction

**Figure 1:**
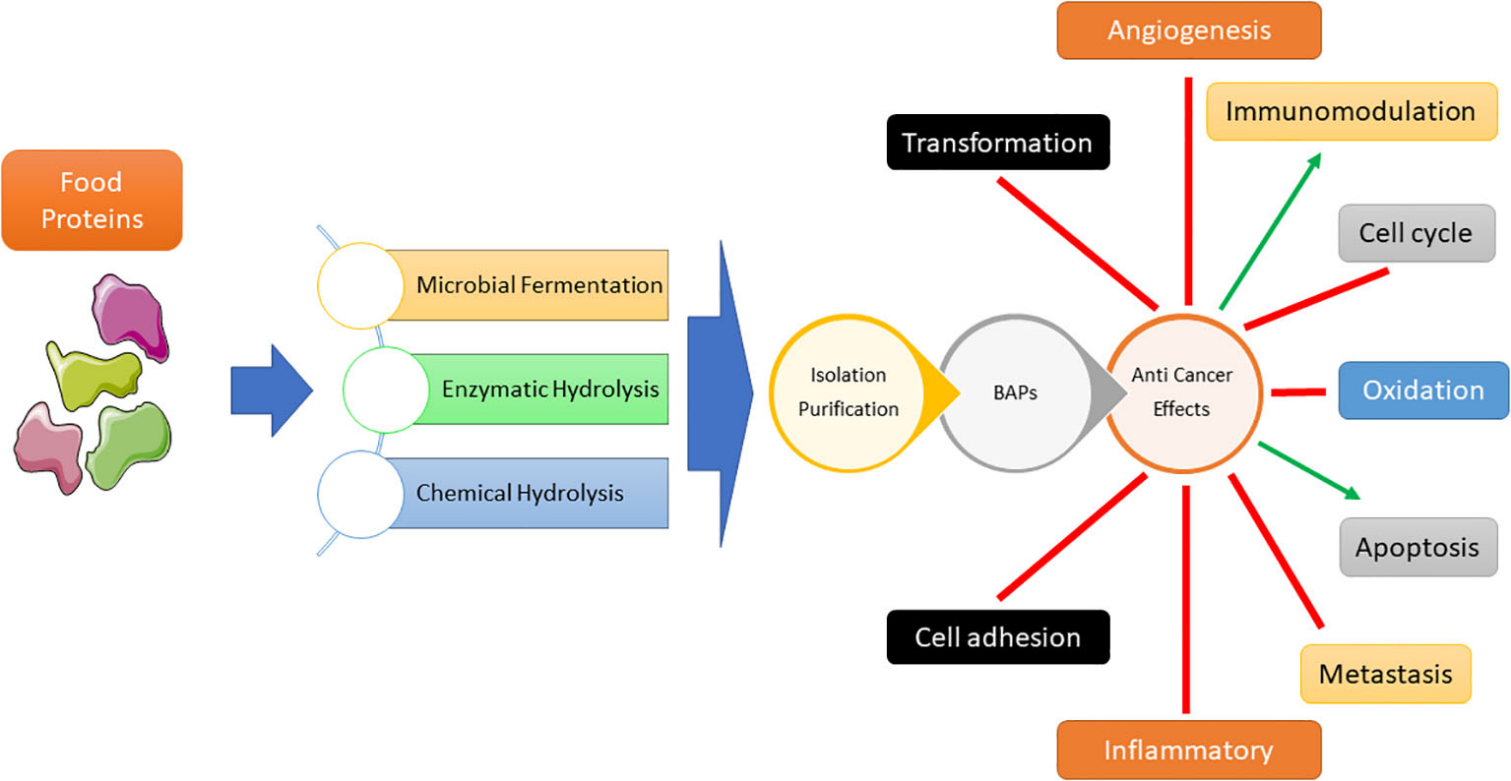
Bioactive peptides (BAPs) production processes and their impacts on various cellular events leading to anti-cancer effect.

Cancer treatments vary depending on tumor type and stage, with chemotherapy, radiation therapy, and surgery being the primary approaches for reducing related mortality. Cancer poses a huge global public health challenge that necessitates substantial attention and allocation of resources (1, 2). Based on a recent cross-continental study conducted in 21 countries, it has been found that cancer is the leading cause of death in many countries (3). One of the primary obstacles in treating this disease is the development of multidrug resistance, wherein cancer cells become resistant to numerous drugs. Despite the significant improvements in the field, there is still a pressing need for the development of more effective and tailored treatments (4). A crucial component of cancer treatment is the precise delivery of chemotherapeutics to cancerous cells, to enhance the treatment efficacy while avoiding adverse effects on healthy tissue (5, 6). “Bioactive peptide” (BP) are organic substances linked by amino acids with peptide covalent bonds. Most BPs are inactive in the main protein and are released after enzymatic processes. Some BPs are also prepared by chemical synthesis. BPs play an important role in human health by influencing different body organs and are considered as a new generation of biologically active regulators (7). The potential anticancer effects of bioactive peptides have garnered significant attention. Peptide-based methodologies present numerous benefits in the realm of cancer therapy, such as heightened selectivity, diminished toxicity towards healthy tissues, and adaptability in the targeting of diverse molecular pathways implicated in the progression of cancer (8–11). A broad variety of engineered and natural peptides have been intensively investigated, encompassing several therapeutic domains. Therapeutic peptides can function for several purposes, including growth factors, hormones, neural transmitters, ion channel compounds, and anti-infective drugs. Cell membrane receptors possess a remarkable level of both affinity and specificity, allowing them to efficiently attach to ligands and subsequently trigger specific intracellular reactions. Therapeutic peptides demonstrate similarities in their mechanism of action to biological ligands and antigens, such as antibodies and therapeutic proteins, hence providing focused and precise therapeutic strategies. In comparison with antibodies for example, despite some limitations including reduced half-life due to rapid excretion and susceptibility to enzyme degradation, they showed clear advantages including cost-effectiveness, extensive tissue penetration, effective cellular internalization, decreased immunogenicity, reduced toxicity to the bone marrow and the liver, and their amenability to chemical modification (12, 13). In the scenario of their use in cancer treatment, bioactive peptides were extensively texted for their ability to induce apoptosis, representing a common strategy to decrease cancer cell proliferation (14). Bioactive peptides in the context of immune modulation, can enhance or suppress immune responses, making them valuable tools for immune modulation (15). They can induce proliferation or activation of immune cells and cytokines, promoting a robust immune defense against pathogens or cancer cells. Additionally, bioactive peptides can inhibit cell migration by influencing cell adhesion, chemotaxis, and tissue remodeling processes (16). This property is particularly important in preventing the migration of cells associated with diseases like cancer, where metastasis is a significant concern (**Figure 1**) (17). Overall, bioactive peptides offer a promising avenue for therapeutic interventions, harnessing the body’s immune system and preventing unwanted cell migration for improved health outcomes (18, 19). This review scope encompasses the most recent research on the anticancer and immunomodulatory properties of bioactive peptides derived from natural sources.

## 2 Peptides with anticancer function

Bioactive peptides have demonstrated several anti-cancer effects on well-established cancer cell lines, including the inhibition of cell migration, suppression of angiogenesis, antioxidant properties, inhibition of cell proliferation, induction of apoptosis, and cytotoxicity (13). The consolidated information can be found in **Table 1**. Altered peptides augment the efficacy of cancer treatment, resulting in enhanced activity of antigen-presenting cells (APCs) for pharmaceuticals and immunizations and the primary objective of the development of modified anticancer peptides (ACPs) is for clinical against cancer. The use of natural biomaterials such as protein hydrolysates or peptides is considered an option in cancer treatment, mainly due to their cost-effectiveness and safety for human health. Peptide-based drugs and vaccines represent a valuable class of therapeutics due to their high permeability, high selectivity, and easy modification (33). They also play an important role in preventing cancer by regulating various genetic pathways. The potential of food protein hydrolysates and peptides as drugs of anti-inflammatory origin has been fully evaluated by *in vitro* and *in vivo* experiments at various levels. Additionally, many anti-inflammatory peptides have been tested as drugs and vaccines in phase I/II clinical trials (33). For example, dTCAPF, a novel hormonal peptide that enters cells through the toll/interleukin-1 receptor, has been shown to be safe and effective in treating patients with prostate cancer, liver cancer/metastatic cancer. Its anticancer activity appears to be associated with the inhibition of angiogenic factors, induction of anticancer cells activity and proliferation, and endoplasmic reticulum stress (34). The relationship between antigenic peptides and correlation structure showed that most antigenic peptides have short segments of 3 to 25 amino acids. Shorter peptides allow greater molecular mobility and expansion and can interact with cancer cells more effectively. Moreover, the antimicrobial activity of peptides is also affected by their amino acid composition, segment, length, total charge, and hydrophobicity (35). Hydrophobic amino acids including Ala, Leu, Pro, Gly and the presence of some specific amino acids such as one or more residues of Arg, Lys, Tyr, Thr, Glu, and Ser are believed to be involved in the selective attack to cancer cells and potent cytotoxic activity (20, 35) because they increase interactions between the peptide and the outer leaflet of cancer cells’ membrane bilayer containing high phospholipid contents (20). It has also been reported that the amount of heterocyclic amino acids greatly enhances the immunity of food peptides (35). However, the exact molecular mechanism of the anti-cancer effect is not clear so far, but many studies have shown that the anti-inflammatory effect of food protein hydrolysates or peptides is associated with the induction of apoptosis of cancer cells. Cell cycle arrest, permeabilization/cell membrane damage, inhibition of cell adhesion, inhibition of topoisomerase, immune system, and intracellular signaling in cancer cells are other possible mechanisms (20, 35).

**Table 1:** Bioactive peptides with anticancer effects.

Product	Bioactive Components	Function	Reference
**Raja porosa (skate)**	FIMGPY (726.9 Da)	Antiproliferative activity by induction of apoptosis on HeLa cell line	(20)
**Phaseolus vulgaris**	GLTSK (505.48 Da), LSGNK (518.29 Da), GEGSGA (521.22 Da), MPACGSS (656.01 Da) and MTEEY (671.98 Da)	Inhibition of cell growth and modification of expression of cell cycle regulatory proteins p53, p21, cyclin B1, BAD on HCT-116, RKO and KM12L4 (human colon cancer cell lines)	(21)
**Seaweed (Eucheuma serra)**	Lectins	Anticancer effect by cytotoxicity, apoptosis, and inhibition of tumor growth	(22, 23)
**Soybean**	Peptied fractions: < 5 kDa, 3–5 kDa, 1–3 kDa, >1 kDa	Antimicrobial, antioxidant, and antitumor functions	(24, 25)
**Amaranth**	VW, GQ/PYY, RWY, WY, RW PWW, PWR, PW, PWY WYS/VGECVRGRCPSGMCCSQF GYCGKGPKYCG	Anticancer, antioxidant and antimicrobial functions	(26, 27)
(28)	GLTSK, LSGNK, GEGSGA, MPACGSS, MTEEY	Anticancer	(29)
**Whey protein hydrolysate**	Lactoferricin	Anticancer and antimicrobial activity	(30)
**Crocodylus siamensis(fresh** **water crocodile)**	NGVQPKYKWWKWWKKWW (2.433 kDa) and NGVQPKYRWWRWWRRWW (2.545 kDa	Induction of cell death by apoptosis on HeLa and CaSki (cervix) cancer cell lines	(28)
**Capra aegagrus** **hircus (goat)**	8 kDa	Cell grows inhibition on HCT-116 (human colon cancer cell line)	(31)
**Telligarca granosa** **(Blood clam)**	WPP (398.44 Da)	Cytotoxicity and change of PC-3 cells morphology on PC-3, DU-145, H-1299 and HeLa cell lines	(32)

### 2.1 Anticancer peptides from plant proteins

Anticancer peptides can be generated by rice and soy protein hydrolysis, specifically through the process of alcalase digestion of rice bran proteins. This discovery established a fundamental basis for the potential utilization of these protein hydrolysates in cancer prevention strategies (36). A study has reported the identification of five peptides (GLTSK, LSGNK, GEGSGA, MPACGSS and MTEEY) obtained from a common bean that exhibited antiproliferative properties on human colon cancer cell lines (HCT-116, RKO, and KM12L4). The peptides were found to affect the activity of enzymes regulating cell proliferation, consequently inducing cell cycle arrest and further apoptosis (21). An ACP with a molecular weight of 1.155 kD, derived from chickpeas, hydrolysis by Flavorzyme has been demonstrated to possess anti-proliferative properties in breast cancer cell lines, specifically MDA-MB231 and MCF-7. This effect is achieved by upregulating the expression of the P53 protein (37), usually activated by different cellular stresses (i.e., DNA damage), and regulating cell cycle progression and death pathways. The peptide sequences SSDEEVREEKELDLSSNE and KELPPSDADW, which were investigated in a study conducted by Karami et al. and obtained from wheat germ protein, had cytotoxic effects on A549 lung cancer cells. The IC50 values for these peptides were measured to be 2.34 μM and 7.25 μM, respectively (38). M. Li et al., reported the dose- and time-dependent cytotoxic activity of papain-hydrolyzed mung bean protein against hepatocellular carcinoma (HepG2) cells with an IC value of 2.99 mg/mL. The authors confirmed that peptides isolated from mung bean protein (Val-Glu-Gly, Pro-Gln-Gly, Leu-Ala-Phe, and Glu-Gly-Ala) could induce apoptosis in hepatocellular carcinoma (HepG2) cells. While high doses may stop cell cycle at the G0/G1 phase and inhibit its progress to the S phase, low concentrations suppress the cell growth during S phase (39). Similarly, glutamic acid from pepsin/trypsin-digested germinated soybeans also showed dose-dependent *in vitro* antiproliferative effects on Caco-2, HT-29, and HCT 116 human cancer cells (40). Peptides extracted from black soybean, mung bean, and adzuki bean have demonstrated the ability to inhibit cancer cells within a concentration range of 200-600 µg/ml (41). Zheng et al. in their study documented the utilization of alcalase and trypsin for the enzymatic hydrolysis of D. catenatum to extract nine distinct peptide fractions. Among these fractions, it was noted that fraction A3 exhibited the highest level of antiproliferative activity against liver (HepG-2), gastric (SGC-7901), and breast (MCF-7) cancer cell lines and the development inhibition rate of cytotoxicity reached a peak of 70%. Notably, the A3 fraction contained three peptides that were abundant: RHPFDGPLLPPGD (1.416 kDa), RCGVNAFLPKSYLVHFGWKLLFHFD (2.994 kDa), and KPEEVGGAGDRWTC (1.504 kDa), and displayed significant pro-apoptotic effects (42). Bioactive peptides derived from Phaseolus vulgaris, soybean meal, amaranth, and seaweed have demonstrated anti-cancer, antioxidant, and cytotoxic properties (22, 24–27, 29), showing potential for complementary utilization in conjunction with conventional cancer therapies. Lunasin is a bioactive peptide derived from soybean or wheat, has been the focus of extensive studies exploring its potential anti-cancer properties (30). Other soy protein peptides exhibit anticancer properties, although their potency is comparatively lower than that of Lunasin (43). The anticancer properties of Lunasin are contingent upon its unique amino acid sequence, which encompasses Arg-Gly-Asp for cellular adhesion and the bending of the polyaspartate acid chain consisting of nine aspartic acid residues. Research findings indicate that Lunasin exhibits promising potential as an adjuvant therapy for cancer and may serve as an effective agent against inflammation, tumor growth, and metastasis in various cancer types (44, 45). Lunasin may potentially serve as a chemo preventive agent in mitigating the incidence of colorectal cancer, breast cancer, and other related malignancies. This function can be executed via various modes and has the capability to impede the interaction between adipocytes and neoplastic cells. Adipocytes, or fat cells, are essential for energy storage, hormone regulation, and overall metabolic health. They play a role in obesity-related health issues and are central to understanding conditions like diabetes and cardiovascular disease. Neoplastic cells, on the other hand, are cancer cells and are crucial in cancer research and treatment. Studying neoplastic cells informs early detection methods, advances in personalized cancer therapies, and strategies for cancer prevention. Both adipocytes and neoplastic cells have far-reaching implications for public health and medical progress, with adipocytes impacting metabolic health, and neoplastic cells being pivotal in the fight against cancer (46, 47). The incorporation of Lunasin into the rice genome yields Lunasin-rich rice, which is anticipated to serve as a functional food for cancer patients (48). The potential integration of Lunasin into the rice genome has been acknowledged as a promising strategy for developing useful food options targeted at individuals affected by cancer.

### 2.2 Anticancer peptides from animal proteins

In a recent study, two short peptides Trp-pro-pro and Gln-Pro have been isolated from the protein hydrolysate of blood clam (Tegillarca granosa) muscle through a combination of ultrafiltration and successive chromatographic techniques. The authors demonstrate that Trp-Pro-Pro might be employed to counteract the surplus generation of reactive oxygen species (ROS) during oxidative stress situations and mitigate the risk of cancers resulting from the accumulation of excessive free radicals within cells. The peptides demonstrated noteworthy cytotoxic properties against diverse cancer cell lines, namely PC-3 (human prostate), DU-145 (human prostate), H-1299 (lung), and HeLa (cervical), in a dose-dependent fashion. Additionally, the treatment with the peptides resulted in notable morphological changes in PC-3 cells (32). Theansungnoen et al. conducted an experiment on cervix cancer cell lines, specifically HeLa and CaSki. They examined the effectiveness of two peptides KT2 (NGVQPKYKWWKW) and RT2 (NGVQPKYRWWRWWR RWW), obtained from freshwater crocodile, and found that they can cause the death of HeLa cells (28). Su et al. in their research involving the utilization of 8 kDa anticancer peptides derived from goat spleen, have shown a notable suppression in the proliferation of HCT116 cells (a type of human colon cancer cells) following a treatment duration of 4-6 days. Furthermore, it was observed that these particular anticancer peptides increased cancer cell apoptosis after a 6–12-hour treatment. This apoptosis induction happened by upregulating the expression of poly (ADP-ribose) polymerase (PARP) and p53 and downregulating the expression of Mcl-1 (31). Chalamaiah et al. discovered that protein hydrolysates (PH) derived from rohu eggs had antiproliferative properties. The study conducted hydrolysis using pepsin on a colon cancer cell line called Caco-2. The findings indicated that the pH had an inhibitory effect on Caco-2 cells, with the strength of the effect increasing with the dosage (23). Chi et al. extracted two peptides from the muscle of the blood clam. One of the peptides was identified and its sequence was determined. WPP (398.44 Da) exhibited potent cytotoxic effects on PC-3 (human prostate), DU-145 (human prostate), H-1299 (lung), and HeLa (cervical) cancer cell lines in a dose-dependent manner. Furthermore, WPP induced significant morphological changes in PC-3 cells also WPP demonstrated the ability to eliminate excessive reactive oxygen species (ROS), thereby preventing the formation of cancers caused by an abundance of free radicals (32). Milk proteins have also been documented as exceptional reservoirs of anticancer peptides. An analysis of Himalayan cheese fermented with probiotic strains of Lactobacillus plantarum NCDC 012, *Lactobacillus casei* NCDC 297, and Lactobacillus brevis NCDC021 has revealed significant *in-vitro* anticancer activity on various cancer cell lines, including human breast cancer (MCF-7), colon cancer cells (HCT-116), transformed human embryonic kidney cells (HEK-T), and neuroblastoma (IMR-32). This activity is primarily attributed to the production of bioactive peptides during fermentation with the added probiotics (49). Ayyash et al. (2018) examined the anti-proliferative effects of camel and bovine milk that had been fermented by probiotic strains of *L. acidophilus* DSM9126 and *Lactococcus Lactis* KX881782. The research group discovered that camel milk, when fermented with Lc. Lactis KX881782, had stronger anti-proliferative effects against cervical cells (HeLa), colon cancer cells (Caco-2), and MCF-7 cancer cells compared to bovine milk. The reduction of proliferation exhibited a robust positive association with the proteolytic activity and DPPH scavenging ability of camel milk that was fermented with *L. acidophilus*, which is accountable for its anti-cancer properties (50). In a study conducted by Yang et al. researchers found that when roe protein from Epinephelus lanceolatus was broken down using protease N, the resulting hydrolysates showed antiproliferative effects on two human oral cancer cell lines (Ca9-22 and CAL 27). These effects were achieved by inducing apoptosis and halting cell cycle progression at the sub-G1 phase (51). Dolastatin 10 is a short peptide comprising five residues that incorporate non proteogenic amino acids (52). This peptide derived from a marine mollusk demonstrated antimitotic characteristics by inhibiting the polymerization of tubulin, suggesting its potential as an agent for anticancer treatment. Dolastatin 10 is classified among a diverse array of marine-derived bioactive compounds that exhibit antineoplastic properties through the inhibition of microtubule growth (53–55). Much like Hemiasterlins, a class of linear peptide derivatives obtained from a marine sponge, Dolastatin 10 also exhibits these characteristics. Research suggests that Dolastatin 10 and Hemiasterlins share similar functions and display encouraging attributes. Su’s team identified an anticancer bioactive peptide-3 (ACPB-3) (56, 57) derived from the spleens or livers of goats. It demonstrates anti-cancer properties against human gastric cancer cell lines (BGC-823) and gastric cancer stem cells (GCSCs), both in laboratory settings and in living organisms. This includes the ability to limit the proliferation of BGC-823 cells and CD44^+^ cells in a manner that is dose dependent, as well as boosting the tolerance to chemotherapy in mice. ACBP-3 also inhibits tumor growth in living organisms (57, 58). Guha and his colleagues discovered TFD-100, a 100 KDa Thomsen-Friedenreich (TF) glycopeptide containing a TF disaccharide (TFD) which can bind to galactin-3, a lectin that specifically binds to β-galactosides. Furthermore, TFD-100 has been shown to prevent the adhesion of androgen-independent prostate cancer cells (PC3), as well as angiogenesis and galactin-3-induced T-cell death (59). Bioactive peptides obtained from diverse origins possess promising potential as agents for combating cancer. However, additional investigation is necessary to comprehensively elucidate their underlying mechanisms of action and ascertain their prospective clinical utility.

### 2.3 Anticancer peptides from animal venoms

Venoms are enriched reservoirs of bioactive peptides that can be utilized for the creation of novel pharmaceutical compounds. However, due to their toxicity, many venoms necessitate chemical alterations before they can be safely employed, and the intricate procedure requires substantial cost and time. Peptides found in venom can interact with certain biological components. This offers potential for the development of new medications that can target prevalent diseases like cancer and neurological disorders (60). The intricated and expensive endeavor of manufacturing novel medications from toxins necessitates substantial financial commitment, especially throughout the clinical stages of II and III, with no assurance of success (60).

Several biologically active peptides found in scorpion venoms exhibit potential anti-cancer properties both in *in vitro* and *in vivo*. One of these peptides has successfully completed phase I and phase II clinical trials (61, 62). The venoms of snakes, scorpions, spiders, honeybees, and cone snails contain bioactive peptides that show potential as abundant reservoirs of chemotherapeutic agents for various human diseases, including chronic inflammation, autoimmune disorders, and cancer (63–66).

#### 2.3.1 Scorpion venom

Scorpions, the most ancient arthropods on Earth, have a venom apparatus attached to their telson, which they employ to inject venom. Scorpions can be classified into 18 separate groups based on their evolutionary relationships. There are around 1,500 species (67) of families, including scorpions. For centuries, venom has been utilized in traditional medicine (68). However, a comprehensive analysis has been conducted on only <1% of all venoms derived from identified sources among different species of scorpions (69). Guo et al. discovered two linear α-helical peptides, TsAP-1 and TsAP-2, in the venom of the Brazilian yellow scorpion, Tityus serrulatus. These peptides possess antibacterial properties. The peptides were tested and found to have inhibitory effects on the growth of human lung cancer cells, namely a squamous carcinoma cell line (NCI-H157) and a lung adenocarcinoma cell line (NCI-H838). In addition, TsAP-2 exhibits threefold more activity compared to TsAP-1 when tested against an androgen-independent prostate cancer cell line (PC-3), MCF-7 cells, and a human glioblastoma cell line (U251) (70). Ali et al. have discovered a novel chlorotoxin-like peptide (Bs-Tx7) from the venom of the Buthus sindicus scorpion. The activity of the chlorotoxin (ClTx) and CFTR channels (GaTx1) is reduced by 66% and 82% respectively, when inhibited by Bs-Tx7. An investigation of the amino acid sequence of Bs-Tx7 has shown a scissile peptide bond (Gly-Ile) that is targeted by human MMP2, an enzyme whose activity is elevated in malignant tumors. This discovery implies that Bs-Tx7 hinders the growth of tumors by reducing the activity of MMP2 (71). Chlorotoxin (Cltx), a peptide derived from the venom of the scorpion Leiurus quinquestriatus, consists of 36 amino acid residues and contains 4 disulfide linkages and has been discovered to effectively block the entry of chloride ions into glioma cells (72–74). Cltx selectively attaches to glioma cells, blocking chloride channels and decreasing MMP-2 production, while having little impact on healthy cells (75–77) The peptides Scolopendrasin I, II, V, and VII derived from S. subspinipes mutilans have antibacterial and anticancer effects. Specifically, Scolopendrasin V exhibit antimicrobial characteristics by attaching to the surfaces of microbial cell membranes (23–25, 27). Centruroides margaritatus venom, namely MgTX, has been discovered to possess a highly effective and specific inhibitor of the peptidyl K^+^ channel. MgTX, consisting of 39 amino acids, effectively suppress the binding of radiolabeled charybdotoxin to voltage-activated channels in synaptic plasma membranes and greatly hinder the development of A549 cells. Furthermore, it has been discovered that MgTX exhibit a reduction in tumor size when tested on a nude mice xenograft model following exposure to malignant tissue (78). Bengalin, a peptide with anticancer properties derived from the venom of Heterometrus bengalensis Koch, has shown the ability to inhibit cell proliferation in K562 and U937 cells with IC50 values of 4.1 and 3.7 mg·mL^−1^, respectively. Importantly, this effect was specific to cancer cells and did not impact normal human lymphocytes. The mechanism of action of Bengalin involves inducing apoptosis (79). Gonearrestide, a peptide with 18 amino acids and a molecular weight of 2192 Da, was discovered in a library of scorpion venom. This peptide has shown potential as an anticancer agent and was derived from the scorpion species Androctonus mauritanicus. Following *in vitro* screenings and bioinformatics analysis, it was proven to be efficacious against several human cancer cells, while exhibiting negligible cytotoxicity towards erythrocytes and epithelial cells. Validation tests and experiments conducted *in vivo*, ex vivo, and *in vitro* showed that it effectively impeded the proliferation of primary colon cancer cells and solid tumors by arresting the cell cycle in the G1 phase (80).

#### 2.3.2 Spider venom

Spider venom has a diverse array of proteins and peptides, including as enzymes, neurotoxins, and cytolytic peptides, that have an impact on ion channels (81). Latarcin 2a (Ltc2a), a peptide derived from spider venom, exhibits cytotoxic effects on human erythroleukemia K562 cells. It induces plasma membrane instability, leading to blebbing, swelling, and ultimately cell death (82). The peptide lycosin-1, produced from spider venom, successfully hinders the growth of cancer cells in a laboratory setting and reduces tumor growth in living organisms by disrupting cell signaling pathways through the reduction of crucial protein functions (83).

Snake venom. Pereira et al. conducted experiments to assess the toxicity of the peptide on cancer cells both in a laboratory setting (*in vitro*) and in living organisms (*in vivo*). They discovered that a concentration of 5 µg/ml of the peptide was deadly to B16-F10 cells (a type of melanoma cells found in mice), Mia PaCa-2 cells (a type of pancreatic carcinoma cells found in humans), and SK-Mel-28 cells (a type of melanoma cells found in humans) (84, 85). BF-30, an antimicrobial peptide derived from the venom of Bungarus fasciatus, is composed of 30 amino acids. It effectively hinders the growth of B16F10 cells in a dose and time-dependent manner when tested in a laboratory setting. Moreover, it significantly suppresses the development of melanoma in mice carrying B16F10 tumors, without inducing any weight loss (86).

#### 2.3.3 Bee and wasp venom

Melittin (MEL) is a well-studied and widely recognized peptide generated from the venom of the honeybee Apis mellifera. It consists of 26 amino acid residues and is classified as an amphiphilic peptide (87). MEL demonstrates inhibitory effects on a range of cancer cell types in laboratory settings, including leukemia, ovarian, lung tumor, carcinoma, glioma, squamous carcinoma, hepatocellular carcinoma, osteosarcoma, prostate cancer, and renal cancer cells (88–92). However, it is important to note that MEL is toxic to normal cells, underscoring the need for precise delivery methods to achieve optimal outcomes (93, 94). Mastoparan, a peptide consisting of 14 amino acids derived from the venom of Vespula lewisii, refers to a group of amphiphilic cationic polypeptides that exhibit anticancer effects when tested in a laboratory setting (95, 96). However, it is crucial to ensure accurate administration in order to prevent any adverse reactions and make necessary alterations for *in vivo* application (96). Various structural alterations can enhance the pharmacodynamic effects of the *in vivo* parameters of the chimeric Mastoparan (95, 97). Upon targeted delivery to the tumor cells, the Mastoparan selectively triggers mitochondrial permeability transition, resulting in the elimination of cancer cells while sparing normal cells. Additionally, Mastoparans derived from other species of wasps have demonstrated anticancer properties. Both V. crabro and V. analis Mastoparans demonstrate antitumor properties against ovarian cancer cells. A concentration of 100 µM of V. crabro Mastoparan resulted in a significant reduction (about 80%) in the relative survival fold of SK-OV-3 cells. Similarly, treatment with 100 µM of V. analis Mastoparan led to a remarkably low survival fold (30%) of SK-OV-3 cells (98). MP1, a peptide similar to Mastoparan, has been found to selectively eliminate cancer cells, including prostate cancer cell line PC-3 (with an IC50 value of 64.68 µM), bladder tumor cells Biu87 (IC50 = 52.16 µM) and EJ (IC50 = 75.51 µM) (99), as well as multidrug-resistant leukemic cells K562/ADM (IC50 = 26.55 µM) (100). Overall, Mastoparan-related peptides produced from wasps have the potential to be considered as primary compounds for the development of innovative anticancer medications (101).

Despite the promising therapeutic benefits of Mastoparan-related peptides produced from wasps on cancer, their implementation in cancer treatment is hindered by various difficulties. Further enhancement is required to improve the instability towards proteases and anticancer activity of MP1 (102). Hence, chemical alterations and substitutions are necessary to enhance the pharmacological characteristics of anticancer peptides. Following the substitution of the thioamide link, MPI-1 exhibited enhanced anticancer activity (IC50 = 20.3 µM for PC-3, IC50 = 21.6 µM for EJ) and reduced adverse effects in both *in vitro* and *in vivo* settings (103). The synthetic derivatives of Decoralin, which is a naturally occurring antimicrobial peptide (AMP) found in wasps. Oreumenes demonstrates significant anticancer effects on MCF-7 breast cancer cells, with an IC50 value of 12.5 µM (104). Both the restriction of conformation and the precise delivery system enhance the therapeutic impact and decrease the toxicity to cells (105–107). This further emphasizes the significance of modifying the conformation and implementing an effective drug delivery system.

## 3 Immunomodulatory activity of bioactive peptides

### 3.1 Peptides with immunomodulatory function

Peptides and proteins are crucial macronutrients that provide the necessary building blocks for protein synthesis and are recognized as a significant energy source (108). Moreover, proteins and peptides present in food can exhibit diverse biological functionalities. Bioactive peptides are obtained from animal and plant proteins through diverse techniques such as proteolysis within the intestinal tract, enzymatic or chemical hydrolysis, or microbial fermentation (19, 109). Immunomodulatory peptides are a type of bioactive peptides that encompass constituents responsible for the regulation of immune cell activity, cytokine generation, and antibody production (110). The immunomodulatory and anticancer properties of these substances are contingent upon their amino acid composition, sequence, and length, as stated in reference (19). These peptides exhibit varying effects on the cell proliferation, inflammation, and cellular protection or destruction, depending on the specific level of action (111–113). Therefore, due to their unpredictable effects on both innate and adaptive immune cells, immunomodulatory peptides are currently a topic of investigation. Depending on whether they stimulate or repress immunological responses, they can operate as either immunostimulants or immunosuppressants. As a result, they hold potential for therapeutic applications in disease treatment (15, 114). The determination of the mechanisms of immunomodulatory peptides functions supplies new opportunities for cancer treatment through improving the immune system. So, this part provides a brief overview of several bioactive peptides from plant and animal sources have been used to modify the immune responses.

### 3.2 Immunomodulatory action BAPs from plant source

Plants are an excellent source of valuable bioactive peptides with various functions. Previous researchers have investigated the immunomodulatory effects of some peptides from plants that are widely used in the human diet like rice, wheat, and legume. **Table 2** indicates several of these bioactive peptides. Macrophages, as innate immune cells, can phagocyte cancer cells and pathogens. In addition, they can interact with other constituents of the innate and adaptive immune system to augment their activities, like secretion of cytokines and cytotoxicity (128). Therefore, regulation of macrophage’s function by immunomodulators would ameliorate the ability of host immune response against cancer. Wu et al. conducted an evaluation of the immunomodulatory effects of the peptide [Glu-Cys-Phe-Ser-Thr-Ala (ECFSTA)] derived from wheat germ globulin on macrophage RAW 264.7 cells (129). The findings of this study indicated that the peptide ECFSTA could enhance cell phagocytosis function and the secretion of nitric oxide (NO), interleukin 6 (IL-6), tumor necrosis factor α (TNF-α), IL-1β, and reactive oxygen species (ROS) generation by RAW 264.7 cells through activation of toll-like receptor 4 (TLR4) and TLR2 (115). In another investigation, Defatted Wheat Germ Globulin was hydrolyzed with different enzymes, including Alcalase, Neutrase, Papain, Pepsin, and Trypsin. Alcalase-prepared peptides were found to exhibit the greatest immunomodulatory activity in relation to lymphocyte proliferation, phagocytosis, and pro-inflammatory cytokine production in RAW 264.7 cells, as mentioned in a previous study (116). In addition to macrophages, it was reported that wheat peptide can modify NK cells and T cells activities. For example, N. Horiguchi et al. demonstrated an augmentation in NK cell function after further administration of one gram of wheat gluten hydrolysate after each meal for six days in healthy people (117). NK cells play important roles in elimination or control of viral infections and cancer cells. NK cells act against tumor cells with cytotoxic role and also secretion of cytokines, mainly interferon-γ (IFN-γ), to regulate adaptive immune responses (130). Treatment of cell culture supernatants from PHA-stimulated human PBMCs with Alcalase wheat gluten protein hydrolysates (WGPHs) resulted in reduction of Th1 and Th17 cytokines secretion (IFN-γ and IL-17, respectively). Although IL-4 levels were not changed but the ratios of IL-4/IFN-γ, IL-4/IL-17 and IL-10/IFN-γ were significantly increased (118). It was shown that rice protein hydrolysates (RPHs) through trypsin digestion may potentially serve as a source of immunostimulatory peptides on RAW 264.7 cells. The study conducted by Xu Z et al. revealed that the YGIYPR peptide derived from rice protein hydrolysates which stands for Tyr-Gly-Ile-Tyr-Pro-Arg has the potential to enhance the proliferation of RAW 264.7 cells (119). While another study investigated anti-inflammatory effect of rice peptides on RAW264.7 cells. They employed both immuno-prediction and in silico simulation techniques to scrutinize rice peptides with potential immunomodulatory properties. Out of 3630 sequences, it was observed that the amino acid sequences GBP1 (NSVFRALPVDVVANAYR), PEP1 (GIAASPFLQSAAFQLR), LR13 (LLPPFHQASSLLR), and TK17 (TPMGGFLGALSSLSATK) exhibited the greatest affinity towards MHC-II. The immunomodulatory properties of four peptides were verified in LPS-induced RAW264.7 cells and they were found to decrease the secretion NO, IL-6, TNF-α, and IL-1β (120, 131). Various legume proteins contain valuable bioactive peptides (132, 133). Several biological effects of them have been reported, including antihypertensive, antimicrobial, antioxidant, anti-inflammatory, and anticancer properties (134–136). Mung bean and soybean are widely utilized legumes that are rich in polyphenols and antioxidants. The study conducted by Ali, NM et al. aimed to evaluate the immunomodulatory, cytotoxic, and antioxidant properties of both fermented and non-fermented mung bean and soybean. The findings of the study indicated that both fermented soybean and mung bean have the ability to induce splenocyte proliferation and promote the release of serum IL-2 and IFN-γ (121). IL-2 is a pro-inflammatory cytokine that plays a crucial role in enhancing T cell proliferation and promoting the release of interferon-gamma (IFN-γ). IFN-γ is primarily produced by Th1 cells, CD8+ T cells, and NK cells, playing a significant role in stimulating cellular immunity. Consequently, it contributes to the elimination of tumor cells (137). According to Kong et al. hydrolyzed soy protein with different proteases generate peptides with various molecular weight and charge. It was found that alcalase soy protein peptides possess higher positive charge and lower molecular weight. These peptides have the most immune-stimulating property by boosting the phagocytic function of macrophages and manipulation of murine spleen lymphocytes (138). In another study, peptide derived from soybean fraction that underwent peptidase R hydrolysis exhibited mitogenic activity and demonstrated a noteworthy increase in the number of IL-12+ CD11b+, IFN-γ + CD49b+ (NK cell), and IFN-γ + CD4+ (Th1) cells in mouse spleens, as well as the activation of cytotoxic activity in spleen cells against the human erythroleukemia cell line (K562). Furthermore, the analysis of DNA microarray revealed the upregulation of genes associated with innate immune response in Peyer’s patch cells of mice administered with peptides (123). Lunasin is a 43 amino acids peptide derived from soy, which possesses anti-cancer and hypocholesterolemic properties (29). In context of immunomodulation displayed that Lunasin-enriched soybean extract (LES) has the intriguing capability to induce both pro-inflammatory and anti-inflammatory activities within the immune system. This observation comes from a study conducted by Paterson and colleagues. Specifically, when macrophages were exposed to LES, they exhibited increased phagocytic activity and elevated production of NO, IL-6, IL-1β, and IL-10. Furthermore, the study found that Lunasin and LES peptides also stimulated EL4 lymphocytes and prompted the secretion of IL-4, IL-5, and IL-10 cytokines. This suggests that soybean peptides, such as Lunasin and LES, have the remarkable ability to modulate or regulate both the innate and adaptive arms of the immune system. In essence, they possess the potential to modulate immune responses in different conditions (122).

**Table 2:** Immunomodulatory effects of bioactive peptides from plant source.

Product	Enzyme	Peptide	Immunomodulation	Reference
**wheat germ globulin**	Alcalase	ECFSTA	Stimulated phagocytosis function and secretion of NO, IL-6, TNF-α and ROS in RAW 264.7 cells.	(115)
**Defatted wheat germ globulin**	alcalase, neutrase, papain, pepsin or trypsin	**-**	The highest immunomodulation activity in proliferation of lymphocyte, phagocytosis and production of pro-inflammatory cytokines in RAW 264.7 cells was reported with alcalase prepared peptides.	(116)
**wheat gluten hydrolysate**	**-**	Glutamine peptide GP-1	Enhancement of NK cell activity.	(117)
**Wheat gluten protein**	Alcalase	WGPHs	Decreased Th1 and Th17 pro-inflammatory cytokines in PBMCs.	(118)
**Rice protein**	Trypsin	YGIYPR	Enhanced the proliferation of RAW 264.7 cells.	(119)
**Rice protein**	Trypsin	NSVFRALPVDVVANAYR, GIAASPFLQSAAFQLR, LLPPFHQASSLLR TPMGGFLGALSSLSATK	Reduced the secretion of NO, IL-6, TNF-α and IL-1β in LPS-induced RAW264.7 cells.	(120)
**Mung bean and soybean**	fermented and non-fermented	-	Stimulated splenocyte proliferation and release of serum IL-2 and IFN- γ.	(121)
**Lunasin-enriched soybean extract (LES)**	-	–	Increasing phagocytic activity and production of NO, IL-6, IL-1β, and IL-10 and EL4 lymphocyte activation.	(122)
**Soybean protein**	Peptidase R	Fraction	Enhanced the count of mouse spleen IL-12^+^ CD11b^+^, IFN-γ ^+^ CD49b^+^, and IFN-γ ^+^ CD4^+^ cells.	(123)
***Pseudostellaria heterophylla* protein**	-	RGPPP	Increased TNF-α secretion NO, ROS and TLR2 expression in RAW264.7 cells.	(124)
**Chlorella vulgarian**	Ethanol and pancreatic at pH values of 7.5-8.0	Three main peptides of molecular masses between 2 and 5 kDa	Stimulated activation of monocyte and macrophage, humoral and cell mediated immune responses.	(125)
**Salvia*. hispanica* L. seeds**	Pepsin and Pancreatin	Peptide fractions (<1, 1-3, 3-5, 5-10, and >10 kDa)	Decreased of NO, TNF-a, IL-1β and IL-6 on BALB/c peritoneal macrophages.	(126)
**Sunflower protein**	Flavourzyme	YFVP, SGRDP, MVWGP and TGSYTEGWS)	inhibited NFκB and enhanced the CD14 expression and the differentiation of monocytes a dendritic cell phenotype.	(127)

There are some reports from peptide of other less common plants. For example, the utilization of proteins extracted from Pseudostellaria heterophylla can serve as a dietary supplement for enhancing immune system function. The digestion of P. heterophylla protein has yielded a novel peptide induced the TLR2/NF-κB pathway. This peptide has been observed to induce a significant increase in TNF-α production, pinocytosis, and TLR2 expression in macrophages (124). The peptides derived from enzymatic hydrolysate of Coix glutelin, exhibit immunomodulatory properties by inducing proliferation of splenocytes in mice and stimulating the production of NO from Raw264.7 cells in a dose-dependent manner as demonstrated by Ling-Ling. Additionally, it has the potential to inhibit excessive activation of cells triggered by LPS (139). According to a study conducted by Velliquette et al., the anti-inflammatory and immune-modulating properties of peptides derived from sunflower protein with Flavourzyme enzyme has been revealed. These peptides attenuate the activation of NF-κB while enhance the expression of surface markers like CD14 and CD86, which are linked to a dendritic cell (DC) phenotype (127). The activation of monocyte and macrophage, humoral and cell-mediated immune responses, as well as hemopoietin, were observed to be stimulated by peptides derived from Chlorella vulgarian 87/1 (125). In addition, Chan-Zapata et al. have extracted bioactive peptides from the seeds of Salvia hispanica L. that exhibit anti-inflammatory properties. These peptides have been found to significantly reduce the levels of NO, TNF-α, IL-1β, and IL-6, while do not affect the viability of BALB/c peritoneal macrophages (126). Overall bioactive peptides derived from plants can influence immune responses to a variety of situations, potentially promoting both inflammatory and anti-inflammatory actions in diverse contexts as needed. Further research may provide the potential applications of plants bioactive peptides in modulating of immune function in cancer, alleviating autoimmune diseases, and mitigating tissue damage in inflammatory conditions.

### 3.3 Immunomodulatory action BAPs from animal source

Previous *in vivo* and *in vitro* studies have reported that bioactive peptides derived from animal sources have the ability of regulating the immune responses, as evidenced in **Table 3**. Among dietary proteins, fish, milk, egg, and chicken proteins are likely valuable source of bioactive peptides in nutritional perspective. The immunomodulatory activity of peptides found in certain fish families has been observed to potentially result in heightened lymphocyte proliferation and macrophage activity, as well as improved natural killer (NK) cell function and cytokine production (140, 155). The lymphocyte proliferation, NK cell activity, CD4^+^ T helper cell count in the spleen, and the production of Th1 (IL-2, IFN-γ) and Th2 (IL-5, IL-6) cytokines in mice were enhanced by marine oligopeptides (MOP), which are low-molecular-weight peptide compounds derived from Chum Salmon (140). These findings suggest that MOP may help the inhibition of tumor growth or metastasis through the activation T cells and NK cells as main mediators against cancer cells. Wang YK and colleagues showed that oyster hydrolysates effectively inhibited the growth of transplantable sarcoma-S180 in BALB/c mice. Additionally, these hydrolysates were observed to enhance the proliferation of lymphocytes in the spleen, as well as the activity of NK cells and the phagocytic activity of macrophages in mice. Thus, it is possible to utilize oyster hydrolysates as a nutritional supplement for the purpose of tumor therapy, as suggested by previous research (141). In addition, the immunomodulatory activities of peptides derived from Labeo rohita egg - were assessed on both innate and adaptive responses in BALB/c mice by Chalamaiah et al. Trypsin hydrolysates peptides have been found to have a significant impact on splenic CD4^+^and CD8^+^ T cells while pepsin hydrolysate enhanced the production of mucosal IgA, macrophage phagocytose capacity as well as NK cell cytotoxicity. As a result, divers rohu egg protein hydrolysates have the potential to stimulate different immune cells (143). Milk proteins, comprising of approximately 80% casein and 20% whey proteins such as α-lactalbumin, β-lactoglobulin, are considered a significant source of bioactive peptides that possess various health-promoting properties such as anti-microbial, anti-hypertensive, anti-oxidative, and anti-diabetic activities (44, 156). The presence of multiple immunomodulatory peptides that can either suppress or stimulate an immune response has been demonstrated (157, 158). According to Chen et al. the administration of milk-derived peptides at a dose of 200 mg kg^−1^ in mice resulted in the most effective regulation of LPS-induced inflammation. This was achieved through the enhancement of immune activity in the spleen and the regulation of immunoglobulin and cytokine secretion (149). The peptide LFP-20, which consists of 20 amino acids and is derived from porcine lactoferrin, can balance Th1 and Th2 response by regulating the expression of Th1 cytokines (IL-12p70, IFN-γ, TNF-α) and Th2 cytokines (IL-4, IL-5, and IL-6), as well as CD3^+^CD8^+^ T cells and B cells, in cases of immune disorder induced by LPS. Although LPS induced remarkable enhancement in production of TNF-α, IL-6, IFN-γ, IL-12p70, IL-4 and IL-5, treatment with LFP-20 declined LPS-induced cytokine secretion (147). Moreover, research has represented that peptides derived from whey and casein proteins play a significant role in regulating the immune system (159). For example, in a study conducted by Rodrıguez-Carrio et al., it was observed that whey β-lactoglobulin fractions, comprising of short peptides, induced the secretion of TNF-α by monocytes. On the other hand, fractions containing large peptides were found to enhance the Th1 cytokine IFN-γ (144). Certain peptide derived from casein generated by strains of Lactic acid bacteria during the process of fermentation have demonstrated the ability to modulate the immune system. The findings of the study revealed that specific peptide fractions derived from fermented milk were capable of inducing the secretion of the anti-inflammatory cytokine IL-10 in human THP-1 monocytes (146). Another peptide from bovine αS1-casein (residues 142-149) significantly increased IFN-γ secretion from CD8+ T cells (160). Therefore, milk peptides may be beneficial in cancer treatment by modifying host immune responses. The egg white has been shown to contain bioactive peptides that play a crucial role in maintaining the viability of the yolk’s embryo (161). The immunoregulatory impact of egg white peptides (EWP) on RAW264.7 macrophage cells and an immunosuppressive BALB/c mice model was evaluated by Zhang, F. et al. The findings obtained from the *in vitro* experiments indicated that the administration of 100 μg/mL EWP resulted in a significant increase in macrophage activation and the secretion of NO, IL-6, IL-10, and TNF-α in RAW264.7 cells. Additionally, an increase in the production of cytokines such as IL-2, IL-6, IL-10, and TNF-α by splenocytes and peripheral blood leukocytes was observed in mice administered with a dose of 150 mg/kg/day of EWP (148). Another study was conducted to investigate the immunomodulatory impact of peptides derived from Selenium-enriched EWP (Se-EWP) on mice that had been immunosuppressed due to cyclophosphamide administration. The group of mice that received the Se-EWP exhibited elevated levels of white blood cells and serum concentrations of IL-6, IL-2, and TNF-α when compared to the EWP groups like pervious research (162). Hence, it is plausible that EWP could potentially serve as a significant factor in mitigating immune-mediated damages and ameliorating the effects of an immunocompromised condition. The hydrolysates of ovalbumin, lysozyme, and whole egg were found to have an inhibitory effect on lymphocyte proliferation and the generation of IL-10 and IL-13. Additionally, these hydrolysates were observed to reduce the TNF-α production from Th1 cells. Furthermore, they were found to inhibit IgG1-class switching induced by LPS and neutralize the release of ROS (150). Thus, the substance in question possesses the ability to serve various functions, including but not limited to acting as an immunostimulant or immunosuppressant and as a mediator for shifting between Th1 and Th2 responses. Chicken is another common nutrient with bioactive peptides. The immunomodulatory function of chicken collagen peptides is attributed to their ability to regulate the secretion of cytokines that promote inflammation. Zhang et al. reported a decrease in the secretion of IL-6, TNF-α, and soluble intercellular adhesion molecule-1 in mice that had received chicken collagen hydrolysate (151). The pathogenesis of osteoarthritis involves the activation of the inflammatory cascade through the stimulation of various cytokines such as TNF-α, IL-10, IL-8, and IL-1β. The study found that the hydrolysis of trypsin in chicken cartilage resulted in a reduction of these cytokine in papain-induced model rats, leading to an alleviation of osteoarthritis symptoms (152). The silkworm pupa (*Bombyx mori*) protein is rich in valuable peptides. It has been used as food and medicine in East Asian Countries (163). Enzymatic hydrolysates of silkworm have demonstrated antioxidant, antitumor and anti-bacterial effects (164, 165). Assessment of immunomodulatory peptides derived from silkworm pupa indicated the administration of 100 μg/ml of identified hexapeptide (PNPNTN) obtained from the alcalase hydrolysate resulted in a significant improvement of Con A- or LPS-induced splenocyte proliferation. The physicochemical properties of this hexapeptide like length, positive charge and hydrophobicity affect its proliferative role. Therefore, novel immunomodulatory peptides are potential therapeutic agent for enhancing immune cell function in the context of food ingredients (153).

**Table 3:** Immunomodulatory effects of bioactive peptides from animal source.

Product	Enzyme	Peptide	Immunomodulation	References
**Chum Salmon**	Complex protease	Marine oligopeptide	Increased the lymphocyte proliferation, NK cell action, number of CD4+ T helper cells in spleen and the secretion of Th1 and Th2 cytokines in mice.	(140)
**Oyster (*Crassostrea gigas*) hydrolysates**	protease	Fraction	Increased the spleen proliferation of lymphocytes, the activity of NK cells, and the phagocytic activity of macrophages in mice.	(141)
**Salmon pectoral fin**	Pepsin	PAY	Reduce the production of NO, PEG2 TNF-a, IL-1b and IL-6 in RAW264.7 cells.	(142)
**Labeo rohita** **egg**	Trypsin, pepsin and alcalase	Fraction	Enhanced the percentage of splenic CD4+ and CD8+ and NK cell cytotoxicity in BALB/c mice.	(143)
**Milk protein (Whey b-lactoglobulin)**	Trypsin	Fractions	Stimulated TNF-a secretion by monocytes and enhanced IFN*-γ.*	(144)
**α-lactalbumin**		Residues 51-53	Enhanced the phagocytic function of macrophages in murine and humans	
**Milk protein (casein phosphopeptides preparation (CPP-III)**	Trypsin	αs2-casein (1-32) and β-casein (1-28)	Augmented production of IgA, IL-5, and IL-6 in spleen cells.	(145)
**Milk protein (casein-derived peptide)**	Fermentation by Lactic acid bacteria	–	Stimulated IL-10 secretion in human THP-1 monocytes	(146)
**Porcine lactoferrin-derived peptide** **(LFP-20)**	-	KCRQWQSKIRRTNPIFCIRR	Balanced the production of both Th1 (IL-12p70, IFN-*γ*, TNF-*α*) and Th2(IL-4, IL-5 and IL-6)cytokines.	(147)
**Egg white peptides (EWP)**			Enhanced macrophage activation and secretion of NO, IL-6, IL-10 and TNF-α in RAW264.7 cells also, incremented the production of IL-2, IL-6, IL-10 and TNF-α in splenocytes of mice.	(148)
**Selenium-enriched egg white peptides (Se-EWP**	Alkaline–neutral protease	SeCys-Trp-Leu-Glu, Trp-Ser-SeCys, SeMet-Ala-Pro, and SeMet-Leu	The treated Se-EWP mice had higher white blood cells count and serum levels of IL-6, IL-2, and TNF-α.	(149)
**Hydrolysates of ovalbumin, lysozyme and whole egg**	Alcalase	–	Decreased lymphocyte proliferation and generation of IL-10, IL-13 and TNF-a inhibited IgG1-class switching induced by LPS.	(150)
**Chicken collagen peptides**	-	-	Reduced secretion of IL-6, TNF-α, treated with the chicken collagen hydrolysate.	(151)
**The hydrolysis chicken cartilage**	Trypsin	Filtration into CCH-I (molecular weight (MW) > 10 kDa) and CCH-II (MW <10 kDa)	Alleviated osteoarthritis by declining the IL-1β, IL-10, IL-8 and TNF-α levels in papain-induced model rats	(152)
**Silkworm pupa protein**	Alcalase	PNPNTN	ameliorated the splenocyte proliferation	(153)
***Hysterocrates gigas***	–	SNX-482	Triggerd M0-macrophages, increasing costimulatory molecules (CD40, CD68, CD80, CD83, CD86)	(154)

The venoms of spiders represent a valuable source of bioactive peptides. Munhoz J et al. conducted an analysis on the effectiveness of SNX-482, a peptide obtained from the venom of African tarantula Hysterocrates gigas, on bone marrow macrophages. The administration of SNX-482 resulted in the activation of M0-macrophages, leading to an increase in the expression of costimulatory molecules such as CD40, CD68, CD80, CD83, and CD86. Additionally, the expression of checkpoint molecules including PD-L1, CTLA-4, and FAS-L was also upregulated, regardless of the administered dosage. Furthermore, it was observed that there was an augmentation in the anticancer response as a result of the upregulation of CCR4, IFN-γ, GZMB, and PDCD1 genes, as well as the secretion of IL-23 (154). So, peptides extracted from venom are likely to be used as adjuvants for improving immunotherapies of cancers. Finally, since previous studies highlight the immunomodulatory applications of different bioactive peptides from animals, they have the potential to serve as immunostimulatory or immunosuppressive agents for the treatment of various diseases like cancers, either independently or in conjunction with other therapies.

## 4 Bioactive peptides with the modulatory function of cell migration

Cell migration is a fundamental physiological process for developing and maintaining multicellular organisms. It has a crucial role in cancer development. Secondary tumors can be prevented by inhibiting the invasion of cancer cells into healthy tissues. Bioactive peptides are gaining increasing attention for their remarkable potential in regulating cell migration, a critical process in various physiological and pathological contexts. For instance, the tripeptide Arg-Gly-Asp (RGD) has been extensively studied for its role in cell adhesion and migration, particularly in the context of cancer metastasis. RGD sequences are known to interact with integrin receptors on the cell surface, influencing cell behavior and migration patterns. Additionally, the bioactive peptide known as substance P has been shown to play a role in neuroinflammation by modulating immune cell migration. These examples highlight the diverse applications of bioactive peptides in regulating cell migration and underscore their significance in understanding and potentially controlling various biological processes (16, 166). This section aims to provide a concise overview of the bioactive peptides implicated in the regulation of cancer cell migration for readers with an interest in this subject matter.

### 4.1 Bioactive peptides from animal sources

These peptides have been derived from various sources, including elastin-derived peptides (EDPs), venoms, and neuropeptide Y (NPY). EDPs released in the extracellular microenvironment during tumoral remodeling of the stroma stimulate cancer cell migration by interacting with their membrane receptor, ribosomal protein SA (RPSA) (167). In pancreatic ductal adenocarcinoma (PDAC), EDPs have been shown to promote cell migration by stimulating the transient receptor potential melastatin-related 7 (TRPM7) channel in human pancreatic cancer cells (167). Bioactive peptides from animal venoms have also been found to affect cancer cell features such as cell proliferation, invasion, migration, and immune response modulation. These peptides selectively target cancer cells without harming normal cells and have been proposed as potential therapeutics for aggressive and deadly brain tumors like glioblastoma (GB) (168). However, these peptides, sometimes, have negative effects and support tumor progression. For example, Bombesin is a bioactive peptide, originally isolated from the skin of the European fire-bellied toad (Bombina bombina) (169) that plays a role in modulating cell migration, particularly in the context of cancer progression. It is a neuropeptide that is highly expressed and secreted by neuroendocrine cells in prostate carcinoma tissues and is believed to be related to the progression of this neoplastic disease (170). In a study on 3T3 and lung fibroblasts, bombesin was found to stimulate cell migration in a time and concentration-dependent manner (171). These results indicate that bombesin may play a role in the cellular processes that are crucial for the advancement of cancer, specifically spread and migration. Overall, bioactive peptides from animal sources may have undesired effects on cancer cell migration and other cancer-related processes.

### 4.2 Bioactive peptides from plant sources

A study showed that hemp peptides, generated by controlled hydrolysis of hemp proteins, exhibited anticancer properties in Hep3B human liver cancer cells. The treatment with hemp peptides increased apoptosis, reduced cell viability, and reduced cell migration in Hep3B cells without affecting normal liver cells. Increased cellular ROS levels, overexpression of cleaved caspase 3 and Bad, and downregulation of antiapoptotic Bcl-2 were all linked to the anticancer effects of hemp peptides. The study also suggested that the Akt/GSK-3β/β-catenin signaling pathway played a critical role in the anticancer properties of hemp peptides (172). Cyclotides are a family of plant-derived cyclic peptides that exhibit a diverse range of bioactivities, such as antimicrobial, anticancer, and anti-HIV properties. They are known for their unique structure and stability, and there exists a considerable potential for the utilization of this substance in the field of pharmaceuticals. Cyclotides have been shown to target cell membranes, which play a crucial role in their mechanism of action (173). In the context of cancer, cyclotides have demonstrated cytotoxicity against various cancer cell lines. Their high stability, small size, oral bioavailability, and tolerance to amino acid substitution make them an ideal platform for designing peptide-based drugs for cancer treatment (17). Some cyclotides are toxic to cancer cells whereas others can be designed to bind and inhibit particular cancer targets. The toxicity of cyclotides is associated with their ability to target and disrupt lipid bilayers containing phosphatidylethanolamine phospholipids (174).

Further research is needed to better understand the specific mechanisms by which cyclotides modulate cell migration and their potential applications in cancer therapy. However, their unique properties and ability to target cell membranes make them promising candidates for the development of peptide-based drugs for cancer treatment.

Flavokawain is a bioactive compound found in the kava-kava plant (Piper methysticum) and has been shown to possess anti-cancer properties (175). Although there is limited information on the direct role of Flavokawain as a bioactive peptide in the modulation of cell migration, some studies have demonstrated its potential in affecting cell migration and invasion in cancer cells. For instance, Flavokawain A (FKA) has been shown to induce apoptosis in breast cancer cell lines MCF-7 and MDA-MB231 and inhibit the metastatic process *in vitro*. FKA selectively promotes cell cycle arrest in these cell lines and induces apoptosis in a dose-responsive way through the internal mitochondrial route, indicating that FKA’s anti-cancer effect is reliant on the presence of p53 (175). Another study looked at FKA’s ability to protect endothelial cells from the oxidative stress that ochratoxin A (OTA) causes in *in vitro* models. FKA was found to reduce inflammation via NFκB inhibition and enhance the phosphorylation of PI3K and AKT, which could stimulate antioxidant activity and antiapoptotic signaling. in human umbilical vein endothelial cells (HUVECs). Depending on the dose under the oxidative stress induced by OTA, FKA also increased the phosphorylation of Nrf2 and the expression of antioxidant genes, such as HO-1, NQO-1, and GCLC (176). Further research is needed to better understand the specific mechanisms by which Flavokawain modulates cell migration and its potential applications in cancer therapy.

Lunasin is found in soy, legumes, and some cereal grains, known for its potential anti-inflammatory, antitumor, and antimetastatic properties (45). Although there is limited information on its role in modulating cell migration, some studies have explored its effects on cancer cells. In breast cancer, Lunasin has been shown to suppress the migration and invasion of breast cancer cells by inhibiting matrix metalloproteinase-2/-9 (MMP-2/-9) via the FAK/Akt/ERK and NF-κB signaling pathways (45). This implies that Lunasin may compete with integrins in order to bind with the extracellular matrix (ECM), consequently suppressing the integrin-mediated signaling pathway. In colon cancer, Lunasin has been found to inhibit metastasis by interacting with α5β1 integrin, inhibiting FAK/ERK/NF-B signaling, and enhancing the ability of oxaliplatin to stop metastases from spreading (177). The objective of this study was to examine the impact of peptides derived from soybean protein β-conglycinin on the motility of peripheral polymorphonuclear leukocytes in humans. The findings of the study demonstrated that the peptides MITLAIPVNKPGR and MIT elicited the migration of polymorphonuclear leukocytes via a mechanism that is dependent on FPR1. The migration process was impeded by the presence of tert-butoxycarbonyl (Boc)-MLP, which is an inhibitor of FPR. Additionally, prior treatment with pertussis toxin (PTX) also hindered the migration. The research findings indicated the identification of chemotactic peptides for human polymorphonuclear leukocytes, which were derived from endogenous enzyme digests of soybean protein (173).

Numerous bioactive cationic peptides (BCPs) have demonstrated the ability to impede cellular migration in breast cancer cells. As an example, the administration of PR39 exhibited notable suppression of invasion and migration in 4T1 cells, potentially working in conjunction with Stat3 siRNA to effectively hinder cellular proliferation and migration. The P44/42 MAP kinase protein, which is essential for the migration of breast cancer cells, was found to be negatively regulated by the peptides FR8P and FR11P Furthermore, it was observed that the compound MAP-04-03 displayed significant inhibitory properties on cellular migration when administered at a concentration of 5 μM. Specifically, it was able to inhibit approximately 40% of cell migration, as determined by an IC50 value of 61.5 Mm (17, 174).

In summary, the intricate interplay between bioactive peptides and cell migration offers an interesting area of study with potential implications for advancing our understanding of cancer progression and treatment.

## 5 Bioactive Peptides from Complementarity-Determining Regions (CDRs) in Immunoglobulins and their Revolutionary Impact on Cancer Immunotherapy

In historical perspectives, constant regions of immunoglobulins were ascribed primarily supportive roles devoid of direct anti-infective or antitumor attributes. Recent investigations, as exemplified by Polonelli et al., underscore the substantial therapeutic potential residing within immunoglobulins, particularly within the complementarity-determining regions (CDRs) (178). CDRs of antibodies have evolved as pivotal domains yielding bioactive peptides with profound therapeutic implications. The work of L. Polonelli and collaborators highlights the prevalence of bioactive peptides originating from CDRs, indicating that immunoglobulin molecules serve as reservoirs for an extensive array of sequences with potential activities against diverse targets (179). Polonelli et al., employing Cotia’s and Kabat’s rules, systematically scrutinized CDRs and identified a cytotoxic killer peptide (KP) derived from VLCDR1 and the framework sequence, demonstrating efficacy against various microorganisms (180). Subsequent engineering of KP, featuring an N-terminal substitution (A1E), not only augmented its effectiveness but also expanded its cytotoxic spectrum against fungi, protozoa, bacteria, and viruses (181–185). The recognition that peptides from the framework sequences adjacent to hypervariable CDRs exhibited cytotoxicity against Candida albicans broadened the scope of potential therapeutic candidates (180). Furthermore, synthetic peptides mirroring fragments of the constant region (Fc region) in IgG, IgM, and IgA classes exhibited cytotoxicity against diverse microorganisms (186). Extending the exploration to monoclonal antibodies (mAbs), Dobroff et al. investigated the antimicrobial, antiviral, and antitumor activities of synthetic CDR-related peptides, demonstrating inhibitory effects in both *in vitro* and *in vivo* settings (187, 188). A4, a monoclonal antibody raised against B16F10 cells, induced apoptosis in melanoma cell lines and afforded complete protection against tumor growth in murine models (188). This investigation encompassed synthetic peptides derived from A4, particularly the VH CDR3 peptide, exhibiting inhibitory effects on melanoma cells and inducing DNA degradation (188). Additionally, the *in vivo* administration of the IgM antibody A4M manifested anti-angiogenic effects through bioactive peptides derived from its CDRs (188). Travassos and collaborators identified the peptide C7H2, inducing tumor apoptosis and reducing melanoma growth through interaction with β-actin (179). Subsequent research by Arruda et al. validated the apoptotic effect of C7H2 across various human tumor cell lines, unraveling molecular mechanisms involving G-actin polymerization, F-actin stabilization, caspase activation, and chromatin condensation (189).

Travassos’ team has pioneered a paradigm shift in cancer immunotherapy by leveraging the potential of complementarity determining regions (CDRs) from immunoglobulins (Ig) to develop innovative antitumor peptides. The seminal study conducted by Figueiredo et al. (190) represents a noteworthy milestone, aiming to identify CDR-derived peptide sequences with potent antitumor activities and immunostimulatory properties. The investigated peptides, including the C36L1 peptide derived from the light-chain CDR1 sequence, exhibited cytotoxic effects against murine melanoma and human tumor cell lines *in vitro*. C36L1 demonstrated both immunostimulatory and direct antitumor activities by inducing microtubule depolymerization, apoptosis, and modulating the PI3K/Akt signaling axis (191). Figueiredo et al. underscored the immune-dependent nature of C36L1’s antitumorigenic responses, elucidating its capacity to restore immunogenic functions in dendritic cells (DCs) by binding CD74 and disrupting interactions with tumor-derived macrophage inhibitory factor (MIF) (192). This groundbreaking discovery laid the groundwork for the development of peptide-based immunotherapies targeting the MIF-CD74 signaling pathway to restore antitumor immune responses in metastatic melanoma. Azevedo et al. extended this work by combining immune checkpoint therapies with MIF inhibitors, demonstrating enhanced responses to anti-CTLA-4 treatment in resistant melanoma. This combined therapy augmented CD8+ T-cell infiltration, facilitated macrophage M1 conversion, and reprogrammed the metabolic pathway of melanoma cells, offering a strategic approach to enhance immune checkpoint blockade therapy responses (193). The research led by Prof Travassos and collaborators has not only revolutionized cancer immunotherapy but has also paved the way for innovative therapeutic development, underscoring the potential of CDR-derived peptides to transcend the conventional scope of antibodies and provide targeted treatment (190–193).

## 6 Conclusion

Bioactive peptides have emerged as a promising frontier in medicine, poised to transform fields like cancer therapy, immunomodulation, and cell migration. These peptides, sourced diversely, offer versatile solutions to complex health challenges. In cancer treatment, bioactive peptides present a compelling alternative to conventional therapies. They display selective effects on cancer cells, modulate migration, and induce apoptosis, promising enhanced efficacy with fewer side effects. Their immunomodulatory properties offer potential in managing autoimmune disorders and bolstering immunity against diseases, including cancer. These peptides can finely tune both innate and adaptive immune responses, paving the way for innovative therapies. Bioactive peptides also play a role in controlling cell migration, aiding in combating diseases’ spread. Both animal and plant-derived peptides exhibit various mechanisms influencing cell migration, shaping disease progression.

While further research and clinical trials are needed to unlock their full potential and address challenges like stability and delivery, bioactive peptides hold immense promise in advancing medicine. They offer more precise, effective, and personalized treatments, heralding a brighter future in healthcare.

## Author contributions

NG: Writing – original draft. SS: Writing – original draft. MJ: Writing – original draft. ÖT: Writing – original draft. AP: Writing – original draft. ZL: Writing – review & editing. MTA: Writing – original draft. MG-H: Conceptualization, Writing – review & editing.
